# Language and Cognition Interaction Neural Mechanisms

**DOI:** 10.1155/2011/454587

**Published:** 2011-08-24

**Authors:** Leonid Perlovsky

**Affiliations:** Harvard University and Air Force Research Laboratory, Harvard University, SEAS, Cambridge, MA 02446, USA

## Abstract

How language and cognition interact in thinking? Is language just used for communication of completed thoughts, or is it fundamental for thinking? Existing approaches have not led to a computational theory. We develop a hypothesis that language and cognition are two separate but closely interacting mechanisms. Language accumulates cultural wisdom; cognition develops mental representations modeling surrounding world and adapts cultural knowledge to concrete circumstances of life. Language is acquired from surrounding language “ready-made” and therefore can be acquired early in life. This early acquisition of language in childhood encompasses the entire hierarchy from sounds to words, to phrases, and to highest concepts existing in culture. Cognition is developed from experience. Yet cognition cannot be acquired from experience alone; language is a necessary intermediary, a “teacher.” A mathematical model is developed; it overcomes previous difficulties and leads to a computational theory. This model is consistent with Arbib's “language prewired brain” built on top of mirror neuron system. It models recent neuroimaging data about cognition, remaining unnoticed by other theories. A number of properties of language and cognition are explained, which previously seemed mysterious, including influence of language grammar on cultural evolution, which may explain specifics of English and Arabic cultures.

## 1. Linguistics and Mathematical Models

How do language interacts with cognition is unknown. How they function in thinking? Is language just a communication device, or is it fundamental in developing thoughts? Do we think with words and phrases, or do we speak without thinking? If both abilities are important, how do we learn? Which words go with which thoughts? To use just 1000 words for 1000 objects, every kid has to learn correct combinations among 1000^1000^ possible combinations, often without explicit teaching as has been the case for most kids around the world for millennia. Learning abstract ideas is even more difficult. Words and sentences are not used in small sets combined with objects and events exactly fitting the intended meanings. Most objects present in every situation are irrelevant for this situation (say a pattern on the floor is irrelevant for understanding that this room is a lecture hall or a dining room). How do we learn to ignore the irrelevant majority of objects and events and to account for the relevant context? Which neural mechanisms of the brain enable learning language and cognition? After a brief review of existing theories and past difficulties, fundamental mechanisms of cognition are described with their mathematical models that enable to overcome past difficulties. 

For long time, logic dominated thinking of mathematicians and the intuitions of psychologists and linguists. Logical mechanisms are similar for language or cognition; both are based on logical statements and rules. Deficiencies of logic established by the fundamental Gödelian results [1] were not properly accounted for. 

Contemporary linguistic interests in the mind mechanisms of language were initiated by Chomsky [2] in the 1950s. He identified “poverty of stimulus” among the first mysteries about language that science had to resolve the tremendous amount of knowledge needed to speak and understand language; it is learned by every child around the world even in the absence of formal training. It seemed obvious to Chomsky that surrounding language cultures do not carry enough information for a child to learn language, unless specific language learning mechanisms are inborn. These mechanisms should be specific enough for learning complex language grammars and still flexible enough so that a child of any ethnicity from any part of the world would learn whichever language is spoken around. This inborn learning mechanism Chomsky called Universal Grammar and set out to discover its mechanisms. Chomsky emphasized the importance of syntax and thought that language learning is independent of cognition. The idea of inborn or innate language mechanisms is called *nativism*.

Initially the available mathematics of logical rules, similar to rule systems of artificial intelligence, was used by Chomsky's followers. Eventually a new mathematical paradigm in Chomsky's linguistics was proposed in [3], *rules and parameters*. This was similar to model-based systems emerging in mathematical studies of cognition. Universal properties of language grammars were supposed to be modeled by parametric rules or models, and specific characteristics of grammar of a particular language were fixed by parameters, which every kid could learn when exposed to the surrounding language. A recent fundamental change of Chomsky's ideas [4] was called the *minimalist program*. It aimed at simplifying the rule structure of the mind mechanism of language. Language was considered to be in closer interactions to other mind mechanisms, closer to the meaning, but stopped at an interface between language and meaning. Today, Chomsky's nativism still assumes that meanings appear independently from language. Logic is the mathematics of modeling language neural mechanisms. 

Many psychological linguists, however, disagreed with the separation of language and cognition. In the 1970s, cognitive linguistics emerged to unify language and cognition and to explain the creation of meanings. Chomsky's idea about a special module in the mind devoted to language was rejected. Language and cognition use similar mechanisms. It is embodied and situated in the environment. Related research on construction grammar argues that language is not compositional, and not all phrases are constructed from words using the same syntax rules and maintaining the same meanings; metaphors are good examples [5–8]. Neither nativism, nor cognitive linguistics, however, lead to a computational linguistic theory explaining how cognition and language are acquired and meanings are created. 

Evolutionary linguistics emphasizes an importance of evolving language and meanings. Language mechanisms are shaped by transferring from generation to generation [9, 10]. This transferring process was demonstrated to be a “bottleneck,” a process mechanism that selected or “formed” compositional properties of language and connected language to meanings [11–13]. The evolutionary linguistic approach demonstrated mathematically that indeed this bottle-neck leads to a compositional property of language. A small number of sounds (phonemes, letters) can be aggregated into a large number of words. By simulation of societies of communicating agents [14], evolutionary linguistics demonstrated the emergence of a compositional language, still the computationally viable model of interacting language and cognition has not been developed; existing mathematical formalisms face combinatorial complexity.

Many aspects of interacting language and cognition cannot be modeled by existing mathematical techniques. Existing theories of language and cognition do not explain many salient aspects of the unknown human neural mechanisms, remaining mysterious. These mechanisms are addressed here. The proposed model resolves some long-standing language-cognition issues. How the mind learns correct associations between words and objects among an astronomical number of possible associations; why kids can talk about almost everything but cannot act like adults; what exactly are the brain-mind differences? Why animals do not talk and think like people? How language and cognition participate in thinking? Recent brain imaging experiments indicate support for the proposed model.

## 2. Cognition: A Mathematical Model

### 2.1. Top-Down and Bottom-Up Neural Signals

Important properties of perception and cognition are revealed by a simple experiment, properties ignored by most theories [15]. Imagine an object in front of you with closed eyes. Imagination is vague not as crisp and clear as with opened eyes. When eyes are opened, the object becomes crisp and clear. It seems to occur momentarily, but actually it takes about 1/5th of a second. This is a very long time for neural brain mechanisms, hundreds of thousands of neural interactions. Let us also note with opened eyes that, we are not conscious about the initially vague imagination, we are not conscious of the entire 1/5th of a second, and we are conscious only about the end of this process: a crisp, clear object in front of our eyes. This experiment has become easy to explain after many years of research.

Explaining this experiment requires understanding mechanisms of instincts, emotions, and mental representations. Perception and understanding of the world is due to mechanism of mental representations or concepts. Concept representations are like mental models of objects and situations; this analogy is quite literal, for example, during visual perception, a mental model of the object stored in memory projects an image (top-down signals) onto the visual cortex, which is matched there to an image projected from retina (bottom-up signal; for more details see [16]). 

Mental representations are an evolutionary recent mechanism. It evolved for satisfaction of more ancient mechanisms of instincts. Here, “instinct” is a simple inborn, nonadaptive mechanism described in [17]. Instinct is a mechanism similar to the internal “sensor,” which measures vital body parameters, such as blood pressure, and indicates to the brain when these parameters are out of safe range. (More details could be found in [18] and references therein.) An organism have dozens of such sensors, measuring sugar level in blood, body temperature, pressure of various fluids, and so forth.

Instinctual-emotional theory of Grossberg-Levine [17] suggests that communicating satisfaction or dissatisfaction of instinctual needs from instinctual parts of the brain to decision-making parts of the brain is performed by emotional neural signals. Emotion refers to several neural mechanisms in the brain [19]; here, it always refers to the mechanism connecting conceptual and instinctual brain regions. Perception and understanding of the mental models, corresponding to objects or situations that can potentially satisfy instinctual needs, receive preferential attention and processing resources in the mind.

Top-down neural signals projected from a mental model to the visual cortex make visual neurons to be more receptive to matching bottom-up signals, or “primes” neurons. This projection produces the imagination that we perceive with closed eyes, as in the close-open eye experiment. Conscious perception occurs, as mentioned, after top-down and bottom-up signals match. For a while, the process of matching presented difficulties to mathematical modeling, as discussed below.

### 2.2. Logic and Computational Complexity 

Computer intelligence cannot compete with animals [20]. Specific mathematical difficulty of models of perception and cognition during the last 60 years was understood as combinatorial complexity (CC) [21–24]. Learning requires training, and algorithms have to learn objects in their multiple variabilities but also in context. Context is a combination of many objects; these combinations lead to incomputable number of operations. Combinations of 100 objects are 100^100^, a number larger than all elementary particle interactions in the entire history of the Universe.

CC difficulties have been related to Gödelian limitations of logic; they are manifestations of logic inconsistency in finite systems [25–27]. Approaches designed specifically to overcome logic limitations, such as fuzzy logic and neural networks, encountered logical steps in their operations: Training requires logical procedures (e.g., “this is a chair”).

Dynamic logic (DL) was proposed to overcome limitations of logic [21, 25, 28–31]. The mathematical description of DL is given later; here, we describe it conceptually [32]. Classical logic is static (e.g., “this is a chair”), and DL is a process “from vague-to-crisp”, from a vague representation, model, statement, decision, and plan, to crisp ones. DL could be viewed as fuzzy logic that automatically sets a degree of fuzziness corresponding to the accuracy of the learned models.

DL models the open-close eye experiment: Initial states of the models are vague. Recent brain imaging experiments measured many details of this process. Bar et al. [33] used functional Magnetic Resonance Imaging (fMRI) to obtain high-spatial resolution of processes in the brain, combined with magnetoencephalography (MEG), measurements of the magnetic field next to head, to provide a high temporal resolution of the brain activity. The experimenters were able to measure high resolution of cognitive processes in space and time. Bar et al. concentrated on three brain areas: early visual cortex, object recognition area (fusiform gyrus), and object-information semantic processing area (OFC). They demonstrated that OFC is activated 130 ms after the visual cortex but 50 ms before object recognition area. This suggests that OFC represents the cortical source of top-down facilitation in visual object recognition. This top-down facilitation was unconscious. In addition, they demonstrated that the imagined perception generated by the top-down signals facilitated from OFC to the cortex is *vague*, similar to the close-open-eye experiment. Conscious perception of an object occurs when vague projections become crisp and match a crisp image from the retina; next, an object recognition area is activated.

### 2.3. Neural Modeling Field Theory

The mind has an approximately hierarchical structure from sensory signals at the bottom to representations of the highest concepts at top [16, 34]. Here, we describe interaction between two adjacent layers in the hierarchy. We give a simplified description, as if eye retina. Matching mental models in memory to bottom-up signals coming from eyes is necessary for perception; otherwise, an organism will not be able to perceive the surroundings and will not be able to survive. Therefore, humans and high animals have an inborn drive to fit top-down and bottom-up signals, the instinct for knowledge [22, 24, 27]. 

The knowledge instinct (KI) is similar to other instincts in that the mind has a sensor-like mechanism, which measures a similarity between top-down and bottom-up signals, between concept-models and sensory signals, and maximizes this similarity. Brain areas participating in KI were discussed in [35]. That publication discussed similar mechanisms considered by biologists since the 1950s; without a mathematical formulation, however, its fundamental role in cognition was difficult to discern. All learning algorithms have some models of this instinct, maximizing correspondence between sensory input and an algorithm internal structure (knowledge in a wide sense). According to the Grossberg and Levine instinct-emotion theory [17], satisfaction or dissatisfaction of every instinct is communicated to other brain areas by emotional neural signals. Emotional signals associated with the knowledge instinct are felt as harmony or disharmony between our knowledge models and the world [36]. At lower layers of the mind hierarchy, at the level of everyday object recognition, these emotions are usually below the level of consciousness; at higher layers of abstract and general concepts, this feeling of harmony or disharmony could be conscious; as discussed in [19, 21, 37–39], it is a foundation of our higher mental abilities; experimental demonstration of these emotions associated with knowledge is discussed in [40]. A mathematical theory combining the discussed mechanisms of cognition as interaction between top-down and bottom-up signals is summarized below following [21, 41]. 

In a single layer of the mental hierarchy, neurons are enumerated by index *n* = *1*,…,*N*. These neurons receive bottom-up input signals, **X**(*n*), from lower layers in the mind hierarchy. **X**(*n*) is a field of bottom-up neuronal synapse activations, coming from neurons at a lower layer. Top-down or priming signals to these neurons are sent by concept models, **M**_*m*_(**S**_*m*_, *n*); we enumerate these models by index *m* = *1*,…,*M*. Each model is characterized by its parameters, **S**_*m*_. The models *represent* signals in the following sense. Say, signal **X**(*n*) is coming from sensory neurons activated by object *m*, characterized by parameters **S**_*m*_. These parameters may include position, orientation, or lighting of an object *m*. Model **M**_*m*_(**S**_*m*_, *n*) predicts a value **X**(*n*) of a signal at neuron *n*. For example, during visual perception, a neuron *n* in the visual cortex receives a signal **X**(*n*) from the retina and a priming signal **M**_*m*_(**S**_*m*_, *n*) from an object-concept model *m*. A neuron *n* is activated if both a bottom-up signal from lower-layer input and a top-down priming signal are strong. Various models compete for evidence in the bottom-up signals, while adapting their parameters for better match as described below. This is a simplified description of perception. Models **M**_*m*_ specify a field of primed neurons {*n*}; hence, the name for this modeling architecture is *modeling fields* [25].

A mathematical model of the knowledge instinct is maximization of a similarity between top-down and bottom-up signals,

(1) $$L\;=\underset{n\in N}{\prod }\;\underset{h\in H}{\sum }r\left(m\right)l\left(n\;|\;m\right).$$

Here, *l*(*n* | *m*) is a conditional similarity between a bottom-up signal in pixel (sensor cell) *n* and top-down concept representation *m*, given that signal *n* originated from concept-model *m*; the functional shape of *l*(*n* | *m*) often can be taken as a Gaussian function of **X**(*n*) with the mean **M**_*m*_(**S**_*m*_, *n*). Conditional similarities are normalized on objects (or concepts) *m* being definitely present, and coefficients *r*(*m*) estimate a probability of objects actually being present. Similarity *L* accounts for all combinations of signals *n*  coming from any model *m*, hence, the huge number of items *M* ^*N*^ in (1); this is a basic reason for combinatorial complexity of most algorithms. A system could form a new model; alternatively, old models are sometimes merged or eliminated. This requires a modification of the similarity measure (1); the reason is that more models always result in a better fit between the models and data. Therefore, similarity (1) has to be multiplied by a “skeptic penalty function,” *p*(*N*, *M*) that grows with the number of parameters in models M, and the growth is steeper for smaller *N*.

KI maximizes similarity *L* over the model parameters **S**. DL is a mathematical technique maximizing similarity *L* without combinatorial complexity. Its salient property is matching vagueness or fuzziness of similarity measures to the uncertainty of the models. DL starts with any unknown values of parameters **S** and defines association variables *f*(*m* | *n*),

(2) $$f\left(m\;|\;n\right)=\frac{r\left(m\right)l\left(n\;|\;m\right)}{\sum_{m^{\prime }\in M}r\left(m^{\prime }\right)l\left(n\;|\;m^{\prime }\right)}.$$

DL determining the Neural Modeling Fields (NMF) dynamics is given by

(3) $${dS}_{m}/dt=\underset{n\in N}{\sum }f\left(m\;|\;n\right)\left[\frac{\partial \ln\;l\left(n\;|\;m\right)}{{\partial M}_{m}}\right]\frac{{\partial M}_{m}}{{\partial S}_{m}}.$$

When solving this equation iteratively, *f*(*m* | *n*) is recomputed according to (2) after each step using new parameter values, (3). Parameter values are not known initially, and uncertainty of conditional similarities (their variances) is high. So the fuzziness of the association variables is high. In the process of learning, models become more accurate and association variables more crisp, and the value of the similarity increases. The number of models is determined in the learning process. The system always keeps a store of dormant models, which are vague, have low *r*(*m*), and do not participate in parameter fitting, except *r*(*m*). When *r*(*m*) exceeds a threshold, a model is activated; correspondingly, an active model is deactivated when its *r*(*m*) falls below the threshold. In modeling interaction between bottom-up and top-down signals, the NMF-DL is similar to ART [42]; otherwise, it is a very different architecture and algorithm. In particular, it uses parametric models, and it fits multiple models in parallel, while associating bottom-up and top-down signals. 

The process of DL always converges [25], it is proven by demonstrating that at each time step in (3), KI (1) increases; thus, DL and KI are mathematically equivalent. Cultural effects of KI are discussed in [43].

### 2.4. Perception Example

**Figure 1:**
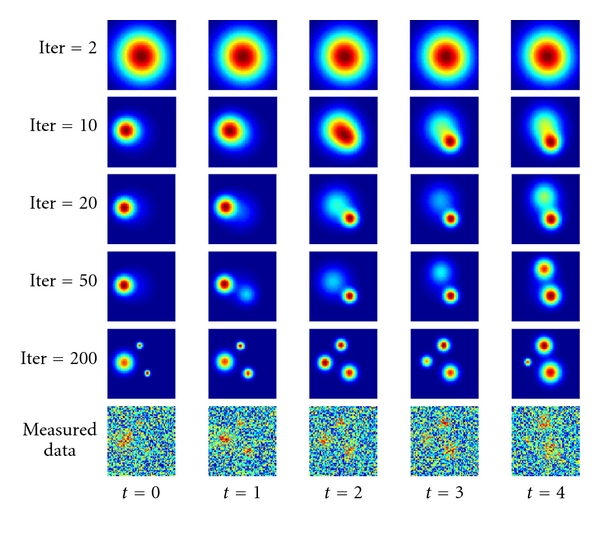
Dynamic logic operation example, finding cognitively related events in noise, in EEG signals. The searched processes are shown in Figure 1 at the bottom row. These events are “phase cones,” circular events expanding or contracting in time (horizontal direction *t*, each time step is 5 ms); in this case, two expanding and one contracting events are simulated as measured by an array of 64 × 64 sensors. Direct search through all combinations of models and data leads to complexity of approximately *M* ^*N*^ = 10^10,000^, a prohibitive computational complexity. The models and conditional similarities for this case are described in details in [44], a uniform model for noise (not shown), expanding and contracting cones for the cognitive events. The first 5 rows illustrate dynamic logic convergence from a single vague blob at iteration 2 (row 1, top) to closely estimated cone events at iteration 200 (row 5); we did not attempt to reduce the number of iterations in this example; the number of computer operations was about 10^10^. Thus, a problem that was not solvable due to CC becomes solvable using dynamic logic.

Below in Figure 1, DL is illustrated with an example described in more details in [44, 45], which demonstrates that DL can find complex process patterns below the noise at about 100 times better than previous algorithms in terms of signal-to-noise ratio [21, 46]. DL solves problems that were previously considered unsolvable, and, in many cases DL converges to the best possible solution of a problem [25, 47–51].

Exact pattern shapes are not known and depend on unknown parameters these; parameters should be found by fitting the pattern model to the data. At the same time, it is not clear which subset of the data points should be selected for fitting. A previous state-of-the-art algorithm for this type of problems, multiple hypotheses testing, tries various subsets [52]. In difficult cases, all combinations of subsets and models are exhaustively searched, leading to combinatorial complexity. In the current example, we use simulated EEG signals of cognitively-related events; as usual, EEG signals are highly noisy, which makes difficult the problem of identifying patterns. The searched patterns are shown in Figure 1 at the bottom row. These events are “phase cones,” circular events expanding or contracting in time (*t*, horizontal direction; in this case two expanding and one contracting events measured by an array of 64 × 64 sensors (each image chip)). Direct search through all combinations of models and data leads to complexity of approximately *M* ^*N*^  = 10^10,000^, a prohibitive computational complexity. 

The models and conditional similarities for this case are described in details in [45], a uniform model for noise (not shown), expanding and contracting cones for the cognitive events. The number of computer operations in this example was about 10^10^. Thus, a problem that was not solvable due to CC becomes solvable using dynamic logic. DL in this example performs better than the human visual system. This is possible due to the fact that the human visual system is optimized for different types of images, not for circular shapes in noise. I would like to emphasize the importance of this example for identifying cognitively related process in the brain EEG. The relevant models used in this example have been originally developed by Kozma and Freeman [53, 54].

### 2.5. Cognition Example

Here, we consider a next higher level in the hierarchy of cognition. At each level of the hierarchy, bottom-up signals interact with top-down signals. For concreteness, we consider learning situations composed of objects. In real brain-mind, learning and recognition of situations proceed in parallel with perception of objects. For simplifying presentation, we consider objects being already recognized. Situations are collections of objects. The fundamental difficulty of learning and recognizing situations is that, when looking in any direction, a large number of objects are perceived. Some combinations of objects form “situations” important for learning and recognition, but most combinations of objects are just random sets, which human mind learns to ignore. The total number of combinations exceeds by far the number of objects in the Universe. This is the reason for this problem having not being solved over the decades [55, 56]. 

**Figure 2:**
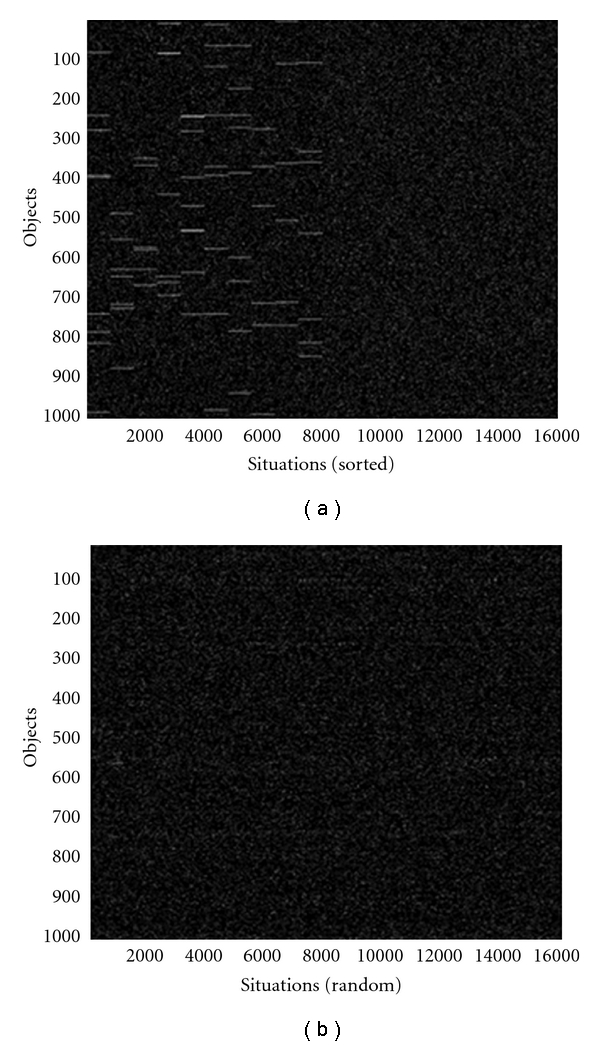
Learning situations; white dots show present objects, and black dots correspond to absent objects. Vertical axes show 1000 objects, and horizontal axes show 10 situations each containing 10 relevant objects and 40 ransom one; in addition, there are 5000 “clutter” situations containing only random objects; (a) shows situations sorted along horizontal axis; hence, there are horizontal lines corresponding to relevant objects (right half contains only random noise); (b) shows the same situations in random order, which looks like random noise.

This example is considered in details in [56]. Here, we summarize the results. The data available for learning and recognition situations in this example are illustrated in Figure 2. Horizontal axes correspond to situations, and the total number of situations are 16,000. Each situation is characterized by objects shown along the vertical axes. The total number of objects is 1000. Objects present in a situation are shown as white pixels, and absent objects are black.  Figure 2(a) illustrates data sorted by situations (horizontal axis). In every “important situation”, there are several objects that are always present in this situation, hence, the white lines in the left part of the figure. In half of situations, there are no repeated objects; these random collections of objects are on the right of Figure 2(a). The same data are shown in Figure 2(b) with randomized order along horizontal line, as various situations actually appear in real life. 

**Figure 3:**
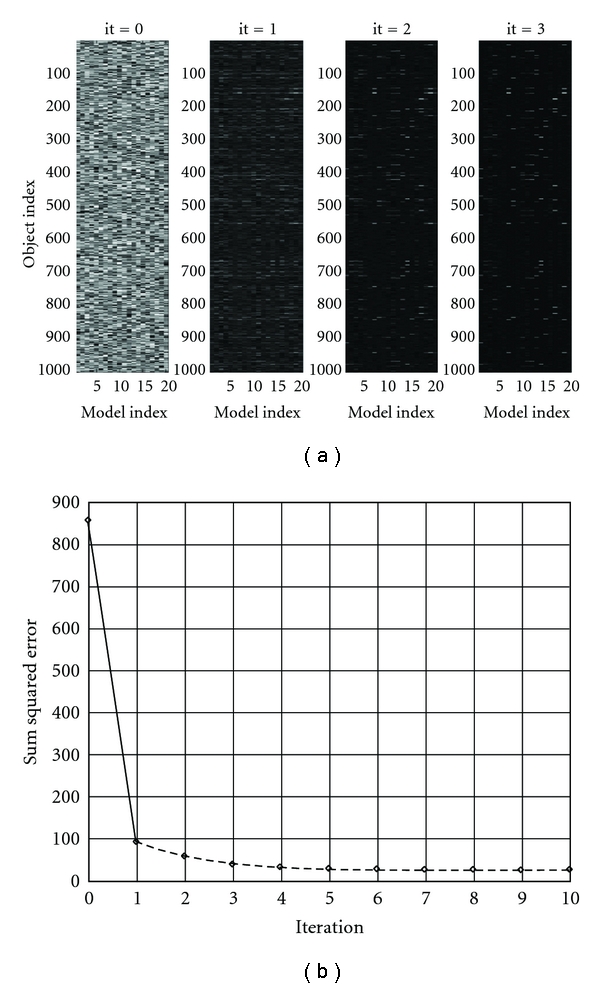
(a) shows DL initiation (random) and the first three iterations; the vertical axis shows objects, and the horizontal axis shows models (from 1 to 20). The problem is approximately solved by the third iteration. This is illustrated in (b), where the error is shown on the vertical error. The correct situations are chosen by minimizing the error. The error does not go to 0 for numerical reasons as discussed in [55].

To solve this problem using a standard algorithm, one can try to sort horizontal axis until white lines appear, similar to Figure 2(a). This would take approximately 10^40,000^ operations, an unsolvable problem. Nevertheless, NMF-DL solves this problem in few iterations, as illustrated in Figure 3.

Figure 3(a) illustrates DL iterations beginning with random association of objects and (arbitrary taken) 20 situations.  Figure 3(b) illustrates that errors quickly go to a small value. The error does not go to 0 for numerical reasons as discussed in [55]. In the above example, relationships (such as on-the-left-of or under) have not been explicitly considered. They can be easily included. Every relation and object can include a marker, pointing what relates to what. These markers are learned the same way as objects [56]. 

The procedure outlined in this section is general in that it is applicable to all higher layers in the mind hierarchy and to cognitive as well as language models. For example, at higher layers, abstract concepts are subsets of lower level ones. The mathematical procedure outlined above is applicable without change.

### 2.6. Language Learning

The procedure outlined in the previous section is applicable to learning language in the entire hierarchy from words up. Phrases are composed of words, and larger chunks of text from smaller chunks of texts can be learned similarly to learning above situations models composed of objects. Grammar rules, syntax, and morphology are learned using markers as discussed above. Lower layer models may require continuous parametric models, like laryngeal models of phonemes [57]. These can be learned from language sounds using parametric models [58–69] similar to a preceding section on perception. 

## 3. The Dual Model of Language and Cognition

Do we use phrases to label situations that we already have understood or the other way around, and do we just talk with words without understanding any cognitive meanings? It is obvious that different people have different cognitive and linguistic abilities and may tend to different poles in the cognitive-language continuum, while most people are somewhere in the middle in using cognition to help with language, and vice versa. What are the neural mechanisms that enable this flexibility? How do we learn which words and objects come together? If there is no specific language module, as assumed by cognitive linguists, why do kids learn a language by 5 or 7 but do not think like adults? And why there is no animals thinking like humans but without human language?

Little is known about neural mechanisms for integrating language and cognition. Here, we propose a computational model that potentially can answer the above questions, and that is computationally tractable, it does not lead to combinatorial complexity. Also it implies relatively simple neural mechanisms, and explains why human language and human cognition are inextricably linked. It suggests that human language and cognition have evolved jointly.

### 3.1. Dual Model

Whereas Chomskyan linguists could not explain how language and cognition interact, cognitive linguists could not explain why kids learn language by 5 but cannot think like adults; neither theory can overcome combinatorial complexity. 

Consider first how is it possible to learn which words correspond to which objects? Contemporary psycholinguists follow the ancient Locke idea, “associationism”: associations between words and object are just remembered. But this is mathematically impossible. The number of combinations among 100 words and 100 objects is larger than all elementary particle interactions in the Universe. Combinations of 30,000 words and objects are practically infinite. No experience would be sufficient to learn associations. No mathematical theory of language offers any solution. NMF-DL solves this problem using the Dual model [70–72]. Every mental representation consists of a pair of models, or two model aspects, cognitive and language. Mathematically, every concept model **M**_*m*_ has two parts, linguistic **M** **L**_*m*_ and cognitive **M** **C**_*m*_:

(4) $$M_{m}=\left\{{ML}_{m},{MC}_{m}\right\}.$$

This dual-model equation suggests that the connection between language and cognitive models is inborn. In a newborn mind, both types of models are vague placeholders for future cognitive and language contents. An image, say of a chair, and the sound “chair” do not exist in a newborn mind. But the neural connections between the two types of models are inborn; therefore, the brain does not have to learn associations between words and objects; which concrete word goes with which concrete object. Models acquire specific contents in the process of growing up and learning, and linguistic and cognitive contents are always staying properly connected. Zillions of combinations need not be considered. Initial implementations of these ideas lead to encouraging results [73–78].

### 3.2. Dual Hierarchy

**Figure 4:**
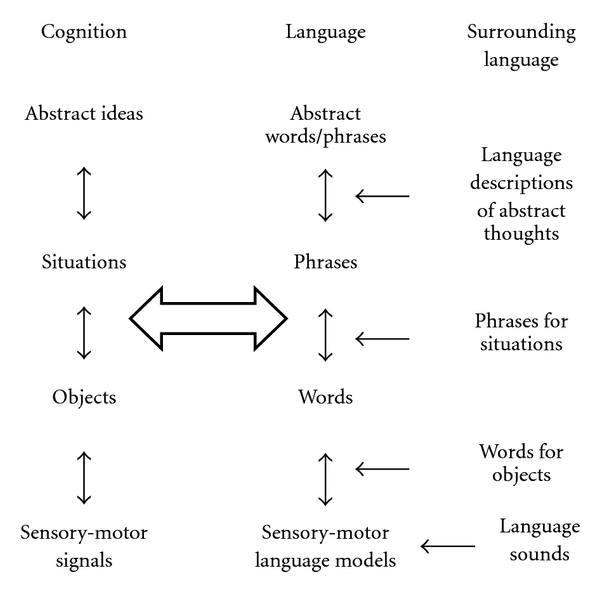
Parallel hierarchies of language and cognition consist of lower-level concepts (like situations consist of objects). A set of objects (or lower-level concepts) relevant to a situation (or higher-level concept) should be learned among practically infinite number of possible random subsets (as discussed, larger than the Universe). No amount of experience would be sufficient for learning useful subsets from random ones. The previous section overcame combinatorial complexity of *learning*, given that the sufficient *information* is present. However, theories of mathematical linguistics offer no explanation where this information would come from.

Consider language hierarchy higher up from words, Figure 4. Phrases are made up from words similar to situations made up from objects. Because of linear structure, language actually is simpler than situations; rules of syntax can be learned similar to learning objects and relations using markers, as described in the previous section. The reason computers do not talk English used to be the fundamental problem of combinatorial complexity. 

Now, that the fundamental problem is solved, learning language will be solved in due course. Practically, significant effort will be required to build machines learning language. However, the principal difficulty has been solved in the previous section. Mathematical model of learning situations, considered in the previous section, is similar to learning how phrases are composed from words. Syntax can be learned similar to relations between objects [55, 71, 79].

The next step beyond current mathematical linguistics is modeling interaction between language and cognition. It is fundamental because cognition cannot be learned without language. Consider a widely held belief that cognition *can* be learned from experience in the world. This belief is naïve and mathematically untenable. The reason is that abstract concepts representations consist of a set of relevant bottom-up signals, which should be learned among practically infinite number of possible random subsets (as discussed larger than the Universe). No amount of experience would be sufficient for learning useful subsets from random ones. The previous section overcame combinatorial complexity of learning, given that the sufficient information is present. However, mathematical linguistic theories offer no explanation where this information would come from.

NMF-DL with Dual model and dual hierarchy suggests that information is coming from language. This is the reason why no animal without human-type language can achieve human-level cognition. This is the reason why humans learn language early in life, but learning cognition (making cognitive representations models as crisp and conscious as language ones) takes a lifetime. Information for learning language is coming from the surrounding language at all levels of the hierarchy. Language model representations exist in the surrounding language “ready-made.” Learning language is thus grounded in the surrounding language.

For this reason, language models become less vague and more specific by 5 years of age, much faster than the corresponding cognitive models for the reason that they are acquired ready-made from the surrounding language. This is especially true about the contents of abstract models, which cannot be directly perceived by the senses, such as “law,” “abstractness,” and “rationality,”. While language models are acquired ready-made from the surrounding language, cognitive models remain vague and gradually acquire more concrete contents throughout life guided by experience and language. According to the Dual model, this is an important aspect of the mechanism of what is colloquially called “acquiring experience.” 

Human learning of cognitive models continues through the lifetime and is guided by language models. If we imagine a familiar object with closed eyes, this imagination is not as clear and conscious as perception with opened eyes. With opened eyes, it is virtually impossible to remember imaginations. Language plays a role of eyes for abstract thoughts. On one hand, abstract thoughts are only possible due to language, on the other, language “blinds” our mind to vagueness of abstract thoughts. When talking about an abstract topic, one might think that the thought is clear and conscious in the mind. But the above discussion suggests that we are conscious about the *language* models of the dual hierarchy. The cognitive models in most cases may remain vague and unconscious. During conversation and thinking, the mind smoothly glides among language and cognitive models, using those that are crisper and more conscious “more available.” Scientists, engineers, and creative people in general are trained to differentiate between their own thoughts and what they read in a book or paper, but usually people do not consciously notice if they use representations deeply thought through, acquired from personal experience, or what they have read or heard from teachers or peers. The higher up in the hierarchy, the vaguer are the contents of abstract cognitive representations, while due to crispness of language models, we may remain convinced that these are our own clear conscious thoughts.

Animal vocalizations are inseparable from instinctual needs and emotional functioning. The Dual model has enabled separation of semantic and emotional contents, which made possible deliberate thinking. Yet operations of the Dual model, connecting sounds and meanings, require motivation. Motivation in language is carried by sounds [80]. Future research will have to address remaining emotionality of human languages, mechanisms involved, emotional differences among languages, and effects of language emotionalities on cultures.

Evolution of the language ability required rewiring of human brain. Animal brains cannot develop ability for deliberate discussions because conceptual representations, emotional evaluations, and behavior including vocalization are unified, undifferentiated states of the mind. Language required freeing vocalization from emotions, at least partially [80, 81]. This process led to evolution of ability for music [81, 81–83]; this is a separate research direction not addressed in this paper.

Another mystery of human cognition, which is not addressed by current mathematical linguistics, is basic human irrationality. This has been widely discussed and experimentally demonstrated following discoveries of Tversky and Kahneman [84], leading to the 2002 Nobel Prize. According to NMF-DL, the “irrationality” originates from the discussed dichotomy between cognition and language. Language is crisp and conscious in the human brain, while cognition might be vague. Yet, collective wisdom accumulated in language may not be properly adapted to one's personal circumstances and, therefore, be irrational in a concrete situation. In the 12th c., Maimonides wrote that Adam was expelled from paradise because he refused original thinking using his own cognitive models but ate from the tree of knowledge and acquired collective wisdom of language [35]. 

The Dual model also suggests that the inborn neural connection between cognitive brain modules and language brain modules is sufficient to set humans on an evolutionary path separating us from the animal kingdom. Neural connections between these parts of cortex existed millions of years ago due to mirror neuron system, what Arbib called “language prewired brain” [85].

The combination of NMF-DL and the dual hierarchy introduces new mechanisms of language and its interaction with cognition. These mechanisms suggest solutions to a number of psycholinguistic mysteries, which have not been addressed by existing theories. These include fundamental cognitive interaction between cognition and language; similarities and differences between these two mechanisms; word-object associations; why children learn language early in life, but cognition is acquired much later; why animals without human language cannot think like humans. These mechanisms also connected language cognition dichotomy to “irrationality” of the mind discovered by Tversky-Kahneman and to the story of the Fall and Original sin. 

The mathematical mechanisms of NMF-DL-Dual model are relatively simple ((2) through (4), also see details in the given references). These mathematical mechanisms correspond to the known structure and experimental data about the brain-mind. In addition to conceptual mechanisms of cognition, they also describe emotional mechanisms and their fundamental role in cognition and world understanding, including role of aesthetic emotions, beautiful, sublime, and musical emotions [80, 82, 83].

### 3.3. Experimental Data

An experimental indication in support of the Dual model has appeared in [86]. That publication has demonstrated that the categorical perception of color in prelinguistic infants is based in the right brain hemisphere. When language is learned and access to lexical color codes becomes more automatic, categorical perception of color moves to the left hemisphere (between two and five years), and adult's categorical perception of color is only based in the left hemisphere.

This provides evidence for neural connections between perception and language, a foundation of the Dual model. It supports another aspect of the Dual model: The crisp and conscious language part of the model hides from our consciousness, the vaguer cognitive part of the model. This is similar to what we observed in the close-open eye experiment: With opened eyes, we are not conscious about vague imaginations.

Another experimental evidence for the Dual model is Mirror Neuron System (MNS) [87]. In humans, primates, and some other social animals, there are neurons that are excited when manipulating objects, and the same neurons are excited, when observing another animal making similar gestures. MNS involves areas of brain near Broca area, where today resides human language ability. Arbib suggested that language system was built on top of the MNS; he called it “language prewired brain” [88, 89]. The Dual model proposed here models this hypothesis: Before language evolves, there are already connections between language and perception/cognition brain areas.

## 4. Language Emotionality, Grammar, and Cultural Evolution

**Figure 5:**
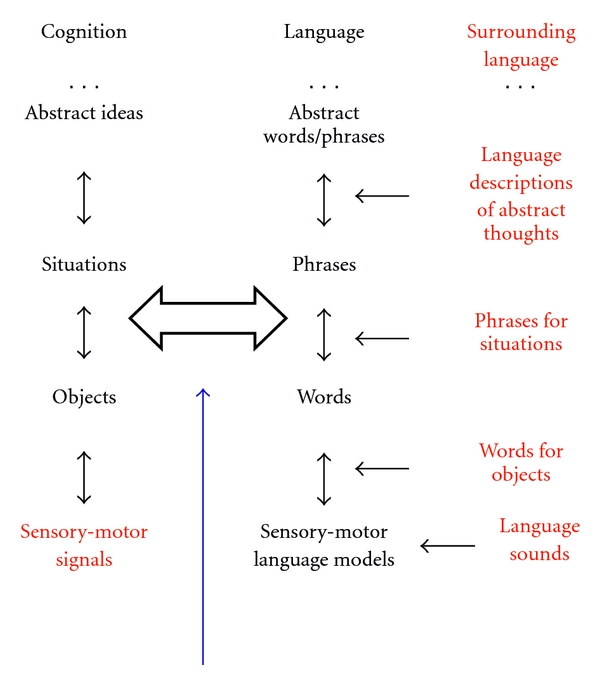
Developing meanings by connecting language and cognition requires motivation, in other words, emotions. If language emotionality is too weak, language is disconnected from the world, meanings are lost, and cultures disintegrate. If language emotionality is too strong, connections could not evolve and cultures stagnate. Is it possible to keep the balance?

Every complex functioning neural mechanism requires motivation, correspondingly, functioning of the Dual model, and requires motivations or emotions, connecting language and cognitive sides of the Dual model, as illustrated in Figure 5.

Emotionality of languages resides in their sounds, like the sound of music moves us emotionally. Animal voicing is fused with emotions; animals lack volunteer control over voice muscles and therefore cannot develop language. Evolution of language required rewiring the brain, so that automatic connection of voice and emotions severed. Language and voice started separating from ancient emotional centers possibly millions of years ago. Nevertheless, emotions are present in language. Most of these emotions originate in cortex and are controllable aesthetic emotions. Emotional centers in cortex are neurally connected to old emotional limbic centers, so both influences, new and old, are present. Emotionality of languages is carried in language sounds, what linguists call prosody or melody of speech. This ability of human voice to affect us emotionally is most pronounced in songs [81]

Emotionality of everyday speech is low, unless affectivity is specifically intended. We may not notice emotionality of everyday “nonaffective” speech. Nevertheless, “the right level” of emotionality is crucial for developing cognitive parts of models. If language parts of models were highly emotional, any discourse would immediately resort to fights and there would be no room for language development (as among primates). If language parts of models were nonemotional at all, there would be no motivational force to engage into conversations, to develop the Dual model. Dual model is fundamental for developing representations of situations and higher cognition [22, 37, 55, 56, 70]. The motivation for developing higher cognitive models would be reduced. 

Primordial fused language-cognition-emotional models, as discussed, have been differentiated long ago. The involuntary connections between voice-emotion-cognition have dissolved with emergence of language. They have been replaced with habitual connections. Sounds of all languages have changed in history, and sound-emotion-meaning connections in languages could have severed. However, if the sounds of a language change slowly, the connections between sounds and meanings persist and consequently the emotion-meaning connections persist. This persistence is a foundation of meanings because meanings imply motivations. If the sounds of a language change too fast, the cognitive models are severed from motivations, and meanings disappear. If the sounds change too slowly the meanings are nailed emotionally to the old ways, and culture stagnates.

These arguments suggest that an important step toward understanding cultural evolution is to identify mechanisms determining changes of the language sounds. These changes are controlled by grammar. In inflectional languages, affixes, endings, fusion, and other inflectional devices are fused with sounds of word roots. Pronunciation sounds of affixes and other inflections are controlled by few rules, which persist over thousands of words. These few rules are manifest in every phrase. Therefore, every child learns to pronounce them correctly. Positions of vocal tract and mouth muscles for pronunciation of inflections are fixed throughout population and are conserved throughout generations. Correspondingly, pronunciation of whole words cannot vary too much, and language sound changes slowly. Inflections, therefore, play a role of “tail that wags the dog” as they anchor language sounds and preserve meanings. This, I think is what Humboldt [90] and Lehmann [91] meant by “firmness” of inflectional languages. When inflections disappear, this anchor is no more and nothing prevents the sounds of language to become fluid and change with every generation. 

This has happened with English language after transition from Middle English to Modern English [92]; most of inflections have disappeared and sound of the language started changing within each generation, and this process continues today. English evolved into a powerful tool of cognition unencumbered by excessive emotionality. English language spreads democracy, science, and technology around the world. This has been made possible by conceptual differentiation empowered by language, not constrained by emotional mechanisms. But the loss of emotionality has also led to ambiguity of meanings and values. Current English language cultures face internal crises, uncertainty about meanings and purposes. Many people cannot cope with diversity of life. Future research in psycholinguistics, anthropology, history, historical and comparative linguistics, and cultural studies will examine interactions between languages and cultures. Initial experimental evidence suggests emotional differences among languages consistent with this hypothesis [93, 94].

Semitic languages and in particular Arabic language are highly inflected. Inflection mechanism called fusion affects the entire word sounds, and the meaning of the word changes with changing sounds; also suffixes control verbs and moods. Therefore, sounds are closely fused with meanings. This strong connection between sounds and meanings contributes to beauty and affectivity of Classical Arabic texts including Quran. On the other hand, creation of new meanings in Classical Arabic is difficult because of this strong connections, remaining unchanged for centuries, and also because of religious restrictions. Arabic language leads to a culture, where meanings and values are strong, but conceptual culture development is slow. There are significant differences between Classical Arabic and street Arabic languages; however, this topic requires separate study. 

Neural mechanisms of grammar, language sound, related emotions-motivations, and meanings hold a key to connecting neural mechanisms in the individual brains to evolution of cultures. Studying them experimentally is a challenge for future research. It is not even so much a challenge, because experimental methodologies are at hand; they just should be applied to these issues. The following sections develop mathematical models based on existing evidence that can guide this future research.

## 5. Future Research

The Dual model implies a relatively minimal neural change from the animal to the human mind. It could emerge through combined cultural and genetic evolution, and this cultural evolution might continue today. DL resolves a long-standing mystery of how human language, thinking, and culture could have evolved in a seemingly single big step, too large for an evolutionary mutation, too fast, and involving too many advances in language, thinking, and culture, happening almost momentarily around 50,000 years ago [95, 96]. DL along with the Dual model explains how changes, which seem to involve improbable steps according to logical intuition, actually occur through continuous dynamics. The proposed theory provides a mathematical basis for the concurrent emergence of hierarchical human language and cognition.

*Solutions* to several principled mathematical problems have been suggested, involving combinatorial complexity. Initial neuroimaging evidence supports the DL mechanism proposed in this paper, and still much remains unknown. DL was experimentally demonstrated for the visual perception; these experiments should be extended to language and interaction of language and cognition. Evolution of languages can be studied using the developed theory and societies of intelligent agents [97].

Mathematical models of some of the mechanisms of evolving languages and cultures have been discussed in [43, 44, 46, 58, 70, 71, 79, 80]. Future research should address evolutionary separation of cognition from direct emotional-motivational control and immediate behavioral connections. Remaining emotionalities of different languages and their effects on cultural evolution shall be addressed.
